# Small Private Key 

PKS on an Embedded Microprocessor

**DOI:** 10.3390/s140305441

**Published:** 2014-03-19

**Authors:** Hwajeong Seo, Jihyun Kim, Jongseok Choi, Taehwan Park, Zhe Liu, Howon Kim

**Affiliations:** 1 Computer Engineering, Pusan National University, Pusan 609-735, Korea; E-Mails: hwajeong@pusan.ac.kr (H.S.); kjhps000@gmail.com (J.K.); bestofcom@gmail.com (J.C.); pth5804@gmail.com (T.P.); 2 Laboratory of Algorithmics, Cryptology and Security, University of Luxembourg, 6 Rue Richard Coudenhove-Kalergi, Luxembourg L–1359, Luxembourg; E-Mail: zhe.liu@uni.lu

**Keywords:** public key cryptography, small private key, multivariate quadratic cryptography, embedded microprocessor, efficient software implementation, ATxmega128a1, AES accelerator, random number generator, signature generation

## Abstract

Multivariate quadratic ( 

) cryptography requires the use of long public and private keys to ensure a sufficient security level, but this is not favorable to embedded systems, which have limited system resources. Recently, various approaches to 

 cryptography using reduced public keys have been studied. As a result of this, at CHES2011 (Cryptographic Hardware and Embedded Systems, 2011), a small public key 

 scheme, was proposed, and its feasible implementation on an embedded microprocessor was reported at CHES2012. However, the implementation of a small private key 

 scheme was not reported. For efficient implementation, random number generators can contribute to reduce the key size, but the cost of using a random number generator is much more complex than computing 

 on modern microprocessors. Therefore, no feasible results have been reported on embedded microprocessors. In this paper, we propose a feasible implementation on embedded microprocessors for a small private key 

 scheme using a pseudo-random number generator and hash function based on a block-cipher exploiting a hardware Advanced Encryption Standard (AES) accelerator. To speed up the performance, we apply various implementation methods, including parallel computation, on-the-fly computation, optimized *logarithm* representation, vinegar monomials and assembly programming. The proposed method reduces the private key size by about 99.9% and boosts signature generation and verification by 5.78% and 12.19% than previous results in CHES2012.

## 1. Introduction

The technology related to embedded systems has made significant progress, making many applications, such as home automation, surveillance systems and environment monitoring services, feasible. However, without secure and robust data protection from security threats, these services cannot be put into practice. To solve these problems, public key cryptography has been studied for several decades. The current main stream is Elliptic Curve Cryptography (ECC), which is an approach based on the algebraic structure of elliptic curves over finite fields. The use of elliptic curves in cryptography was suggested independently by Koblitz [1] and Miller [2] in 1985. The technology provides a short key-size and various applications, including Elliptic Curve Digital Signature Algorithm (ECDSA), Elliptic Curve Diffie-Hellman (ECDH).

The alternative multivariate quadratic ( 

) cryptography provides encryption and digital signatures with modest computational resources [3]. There is no feasible attack to lattice-based cryptosystems that has been discovered yet under a quantum computing environment, while those to factoring for Rivest Shamir Adleman (RSA) and Digital Signature Algorithm (DSA) and Elliptic Curve Cryptography (ECC))-based systems already exist. However, the large size of the public and private keys required makes it difficult to fit such systems into low-cost devices like Radio-frequency identification (RFID) tags and smart-cards.

In this paper, we study an efficient implementation of 

 cryptography in terms of shortening the private key and reducing the computational cost of signature generation and verification. We focus on 

 techniques on an embedded processor, because ECC has been studied for several decades and has reached its technological pinnacle [4]. In contrast, 

 cryptography has only recently begun to receive attention, and there is considerable room to improve its performance. Previously, small public key implementations have been actively studied, but the private key analogue has not. In this paper, we implement small private key 

 cryptography using a Pseudo-Random Number Generator (PRNG). To enhance its performance, we adopt an Advanced Encryption Standard (AES) module for the PRNG and hash function and use optimized techniques, including parallel computation, on-the-fly computation, vinegar monomials, optimized *logarithm*representation and assembly programming.

The paper is organized as follows. In Section 2, we give an introduction to the basic structure of Multivariate Quadratic Public Key Scheme ( 

PKS)and related technologies. In Section 3, we present efficient implementation techniques for embedded microprocessors. In Section 4, we evaluate and analyze the performance of the proposed method. Finally, in Section 5, we conclude the paper with a brief summary of our contributions.

## 2. Related Work

### 2.1. Unbalanced Oil and Vinegar

The idea of Unbalanced Oil and Vinegar (UOV)-signature schemes is to use a public multivariate quadratic map, $\text{P}:{\text{F}}_{q}^{n}\to {\text{F}}_{q}^{m}$, with:

$$\text{P}=\left(\begin{array}{c} p^{\left(1\right)}(x_{1},\ldots ,x_{n})\\ \vdots \\ p^{\left(m\right)}(x_{1},\ldots ,x_{n}) \end{array}\right)$$

and:

$$p^{\left(k\right)}(x_{1},\ldots ,x_{n}):=\underset{1\leq i\leq j\leq n}{\text{∑}}\alpha_{ij}^{\left(k\right)}x_{i}x_{j}=x^{T}\beta^{\left(k\right)}x$$

where *β*^(^*^k^*^)^ is the (*n* × *n*) matrix describing the quadratic form of *p*^(^*^k^*^)^ and *x^T^* = (*x*_1_, …, *x_n_*)*^T^* [5]. The trapdoor is given by a structured central map, $\text{F}:{\text{F}}_{q}^{n}\to {\text{F}}_{q}^{m}$, with:

$$\text{F}=\left(\begin{array}{c} f^{\left(1\right)}(u_{1},\ldots ,u_{n})\\ \vdots \\ f^{\left(m\right)}(u_{1},\ldots ,u_{n}) \end{array}\right)$$

and:

$$f^{\left(k\right)}(u_{1},\ldots ,u_{n}):=\underset{1\leq i\leq j\leq n}{\text{∑}}\gamma_{ij}^{\left(k\right)}u_{i}u_{j}=u^{T}\delta^{\left(k\right)}u$$

In order to hide this trapdoor, secret linear transformation 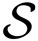 is chosen, such that 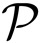 := 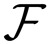 ◦ 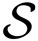 [6]. For the UOV signature scheme, we define two variables called vinegar (*u_i_*, *i* ∈ *V* := {1,…, *υ*}) and oil (*u_i_*, *i* ∈ *O* := {*υ* + 1,…, *n*}). The central map, 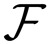, is given by:

$$f^{\left(k\right)}(u_{1},\ldots ,u_{n}):=\underset{i\in V,j\in V}{\text{∑}}\gamma_{ij}^{\left(k\right)}u_{i}u_{j}+\underset{i\in V,j\in O}{\text{∑}}\gamma_{ij}^{\left(k\right)}u_{i}u_{j}$$

The number of vinegar variables is twice the number of oil variables to make the protocol secure. The transformation involves fully mixing the oil and vinegar variables, so that malicious users cannot obtain secret values by separating the oil and vinegar variables.

#### 2.1.1. Signature Generation

To sign a document, *d*, a hash function, $\text{H}:{\text{F}}_{q}^{*}\to {\text{F}}_{q}^{m}$, is used to compute the hash value $h=\text{H}(d)\in {\text{F}}_{q}^{m}$. Next, one computes *y* = 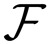
^−1^(*h*) and then *z* = *S*^−1^(*y*). The signature of a document, *d*, is $z\in {\text{F}}_{q}^{n}$.

#### 2.1.2. Signature Verification

To verify the authenticity of a signature, the hash value, *h*, of the corresponding document and the value *h′* = 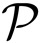
(*z*) must be computed. If *h* = *h′* holds, the signature is accepted; otherwise, it is rejected.

#### 2.1.3. Public Key Optimization

At CHES2011 (Cryptographic Hardware and Embedded Systems, 2011), the 0/1 UOV method for reducing the size of the public key was presented [7]. Choosing the special structure ( 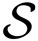, 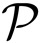), it was reported that the key size and runtime of the verification algorithm could be reduced. The concept behind the reduction of the public key size is the use of a partially cyclic public key and *GF*(2) (Galois Field) elements for coefficients. The method proceeds by generating a partially circulant matrix and then computes the public key transformation matrix from a linear map to compute the corresponding secret key. If the specific requirements are met, we can generate the public key from small-size cyclic keys. Furthermore, coefficients of the *GF*(2) form are easily computable, reducing both the size and verification time.

#### 2.1.4. Private Key Optimization

To reduce the private key size of 

 schemes, we can use a PRNG for key generation. This reduces the key size down to the size of the seed values. Recently, private key generation has been implemented using an RC4-based PRNG [8]. The basic idea is to generate a private key from symmetric cryptography, which can be used as the private coefficients. However, the method is implemented on PCs using JAVAso straight-forward implementation of this method on resource constrained device is infeasible, because PRNG has high overheads for embedded microprocessors. For a light-weight implementation, an embedded encryption module could be exploited. This approach was firstly introduced in INDOCRYPT2012 [9], where sub-operation of the RFSB-509 generating constant is concurrently computable by accumulating previous results. In terms of PRNG, the first implementation of AES-based PRNG was described in [10]. The method exploits AES counter mode of operation to generate high entropy random numbers with high throughput.

### 2.2. Previous Implementations on Embedded Microprocessors

A software implementation of enTTS (20, 28) on a MSP 430 microprocessor was reported in [11]. The signing and verification operations were executed within 71 ms and 726 ms, respectively. At CHES2004, Yang *et al.* reported signs of TTS(20, 28) in 144 ms, 170 ms and 60 ms and TTS(24, 32) in 191 ms, 227 ms and 85 ms for i8032AH, i8051AH and W77E59, respectively [12]. Recently, at CHES2012, implementations of UOV, Rainbow and enTTS on an eight-bit microprocessor were reported. The author implemented 

 signatures with security levels of 2^64^, 2^80^, and 2^128^ and demonstrated the feasibility of such protocols on resource-constrained devices. They also provided specific implementation techniques, such as self-invertible linear maps, LU decomposition and logarithm representation. First, self-invertible linear maps do not, by definition, require inversion, and their private key is smaller than a normal map. Second, LU decomposition factorizes a matrix into the product of lower triangular and upper triangular matrices. The decomposition representation reduced the straightforward implementation cost of Gaussian elimination. Finally, logarithm representation simply performs multiplication on a Galois field by computing addition. Until now, few implementations have been reported on embedded microprocessors, and even private key optimization has not been studied. For this reason, we have focused on a private key reduced model for embedded microprocessors.

### 2.3. Target Platform and Tools

We used ATxmega128a1on an Xplain board as our target platform. This microprocessor has a clock frequency of 32 MHz, 128 KB flash program memory and 8 KB SRAM. Furthermore, the device provides an AES crypto-accelerator that computes the encryption within 375 clock cycles. This is significant progress compared to the software implementation of AES on the ATmega128 processor, which requires 1,993 ∼ 3,766 clock cycles [13,14] with pre-computations. In this paper, we provide a novel signature generation for modern microprocessors that uses an embedded AES accelerator for the PRNG and hash function. These approaches significantly reduce the size of the private key and optimize the computational performance, as well. To use AES module, we should trigger AES operation by following instructions as described in Algorithm 1. First, a status bit for AES operation is set. Second, a key and plain text are allocated to the AES accelerator, and then, we trigger the AES execution, which takes 375 clock cycles. During the execution, we can perform other operations using the microprocessor, because AES operations are independently executed on the AES accelerator. After the execution, we obtain the cipher-text generated from the AES accelerator by accessing the storage address. Our program is written in assembly for the main computations and partly C language for the interface. The development tool is the latest version of Atmel Studio 6.0.



**Algorithm 1:** AES encryption using AES accelerator
Input: Secret key *k*, plain text *p*Output: Cipher-text *c*1.AES accelerator setting2.Move *k* to key storage in AES accelerator3.Move *p* to plain text storage in AES accelerator4.Execute the AES accelerator5.Wait for completion (375 clock cycles)6.Get *c* from cipher-text storage in AES accelerator


### 2.4. Random Number Generator Based on a Block Cipher

Random numbers are widely used as seeds for cryptographic operations and secret key generation. Among various types of random number generators, a block cipher-based random number generator is considered in this paper to exploit the AES module in an embedded board. The following equation outlines the process of random number generation. The notations, *enc*, *C*, *k* and *R*, represent the encryption process, counter (secret seed), secret key and random number stream. First, the counter is encrypted with secret key, *k*. The output is then bitwise XORedwith counter value *C_i_*, and the encryption can proceed. This process is iterated until we obtain a suitable size of random numbers.

(1) $$\begin{array}{r} R_{1}=enc_{k}(C_{1})\\ R_{i+1}=enc_{k}(C_{i}⊕R_{i})\\ \text{where}:C_{i}=C_{i-1}+1 \end{array}$$

The purpose of introducing the block cipher-based random number generator is that we will use it to generate the secret coefficients. This is possible using the AES accelerator on modern embedded boards, which can generate encrypted data conveniently. The AES accelerator is a peripheral device, so it operates independently of the microprocessor. We can order the encryption on the AES accelerator while simultaneously computing a signature on the microprocessor. This method can boost performance and reduce the required program memory. Furthermore, the PRNG process follows cipher-block chaining (CBC); this is efficiently executed using the CBC option on an embedded processor. In our implementation on ATXmega, timing and ROM cost around 40 (clock/bytes) and 204 (bytes), respectively. This result is reasonable for embedded processors. For randomness characteristic, we evaluated our random sequence on the National Institute of Standards and Technology (NIST)-test suite [15]. Firstly we collected a pseudo random number sequence from block cipher-based PRNG by two gigabytes. Then, we operated the NIST statistical test suite version 1.6 with the number of bit streams and the length of the bit set to 100 and 1,024, respectively. The results are described in Table 1, and reasonable proportion rates are achieved.

For the security concern, the strength is based on the bit-length of block-cipher. If the inner state of the generator is compromised once, the adversary could foresee future outputs. To resolve this problem, we should compute random numbers with refreshed seed values. One possible challenge to AES in counter mode would be a timing attack on the value of the counter. We can prevent these attacks by using a counter that always takes the same amount of time to increment its value, and AES-based PRNG could give a periodic generation. This drawback could be solved by reseeding the secret values on proper timing.

### 2.5. Hash Function Based on a Block Cipher

Hash functions compress an input of arbitrary length to a string of fixed length. Our main motivation for constructing a hash function based on a block cipher is to minimize the design and implementation effort. The hash function based on the block cipher is conducted according to Equation (2), where *H*, *p_i_*, *N* and *E* denote the hash code, plain text, nonce and encryption process, respectively. This structure follows the Davies–Meyer single-block-length compression function. The security level of the one-way hash function is determined by the minimum of the size of the key and the block length [16]. To ensure a sufficient security level, we used a 128-bit secret key for the AES-based hash function.

(2) $$\begin{array}{c} H_{1}=enc_{p_{1}}(N)⊕N\\ H_{i}=enc_{p_{i}}(H_{i-1})⊕H_{i-1} \end{array}$$

In our hash function, we exploit the AES-accelerator for the block-cipher-based hash function. Previous 

PKS implementations on an embedded board were not concerned much with hash functions. However hash function should be included for practical purposes, because normally, a message is compressed in its own embedded board, not other places. In Table 2, we can find hash function implementations on the ATmega board. Compared with other results, our result improves speed and size altogether. In the case of speed, we use a dedicated hardware crypto module, so this is faster than other results that implement the functions in software. Furthermore, our result does not use much memory, because we only need to use hardware control code.

The 

PKS implementation consists of two parts, including signature generation and verification. The signature generation produces private coefficients and computes a signature message. In the case of signature verification, the signature is verified by checking the validity of the provided signature. By introducing AES-based PRNG, we significantly reduced the private key size, and with the optimized implementation, we show performance enhancements in both the signature generation and verification parts. The following subsections describe the optimization and implementation methods in detail.

### 2.6. Parallel Computation

**Figure 1.**
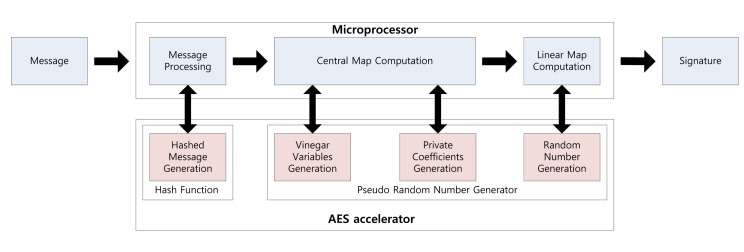
Signature generation process on a microprocessor.

**Figure 2.**
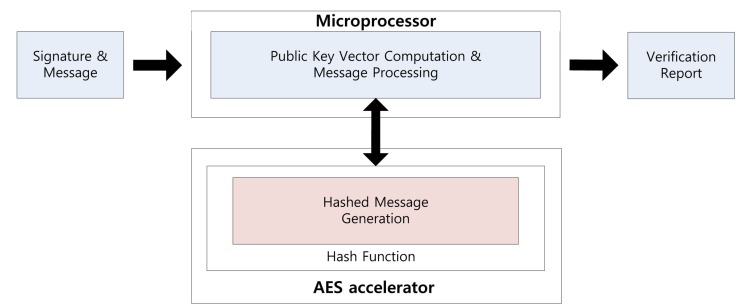
Signature verification process on a microprocessor.

Using the parallel feature of the AES accelerator, we can compute a signature while generating the private coefficients. Signature generation on the embedded processor is described in Figure 1. First, the message is hashed using the hash function. The output is 16 bytes each time and takes 375 clock cycles. Central map computation is then executed, while vinegar variables and private coefficients are generated. These operations are conducted in independent modules, so we can compute both operations together. For this reason, we do not need any additional computation costs to generate the private coefficients. After central map computation, we generate the coefficients of the linear map. As a result of this, the overheads of the key generation process are absorbed in the central and linear map computations. This parallel computation technique can be applied to the verification process for message hashing, which is described in Figure 2. We can the conduct hash function computing the verification process, so one operation is absorbed into other operations.

## Implementation of Small Private Key 

PKS

3.

### 3.1. Logarithm Representation

Multiplication and inversion operations on *GF*(2^8^) can be easily computed using *logarithm* representation in Algorithm 2, which transforms multiplication to a simple addition operation.



**Algorithm 2:** Implementation of Gaussian Elimination.
Input: Coefficients of Gaussian map *g*_(_*_i_*_,_*_j_*_)_, message *m_i_*, where 1 < *i*, *j* ≤ *o*, symbol *o* denotes the number of oil variables, the upper subscript describes the representation transition and the bottom subscript denotes the index. Steps from 1 to 18 describe forward elimination and steps from 19 to 26 describe backward elimination.Output: Result *r_i_* of Gaussian elimination.1.for *i* = 1 to (*o* − 1) do2. for *t* = *i* to *o* do3.   
$\text{temp}\_g^{l}=in\upsilon^{n\to l}(g_{\left(t,i\right)}^{n})$4.  for *k* = *i* to *o* do5.    
$g_{\left(t,k\right)}^{l}=\text{temp}\_g^{l}+\log^{n\to l}(g_{\left(t,k\right)}^{n})$6.    
$g_{\left(t,k\right)}^{n}=exp^{l\to n}(g_{\left(t,k\right)}^{l})$7.  end for8.   
$m_{t}^{\text{l}}=\text{temp}\_g^{l}+log^{n\to l}(m_{t}^{n})$9.   
$m_{t}^{n}=exp^{l\to n}(m_{t}^{l})$10. end for11. for *k* = *i* + 1 to *o* do12.  for *t* = *i* to *o* do13.    
$g_{\left(k,t\right)}^{n}=g_{\left(i,t\right)}^{n}⊕g_{\left(k,t\right)}^{n}$14.  end for15.   
$m_{k}^{n}=m_{i}^{n}⊕m_{k}^{n}$16. end for17.end for18.
$r_{o}^{n}=exp^{l\to n}(\log^{n\to l}(m_{o}^{n})+in\upsilon^{n\to l}(g_{\left(o,o\right)}^{n}))$19.*count* = 120.for *i* = *o* − 2 to 0 do21. for *j* = *o* − 2 to *o* − 1 − *count* do22.   
$r_{i}^{n}=r_{i}^{n}⊕{\text{exp}}^{l\to n}(\log^{n\to l}(r_{j+1}^{n})+log^{n\to l}(g_{\left(i,j+1\right)}^{n}))$23. end for24. 
$r_{i}^{l}=r_{i}^{n}⊕m_{i}^{n}$25. *count* = *count* + 126.end for


To compute multiplication, values are first converted into their *logarithm* form by looking up the *logarithm* table. Then, the relevant values are added, and the sum is returned to a *normal* representation by looking up the *exponential* table.

The representation setting is selected in an optimized way when we generate the private coefficients, vinegar values and coefficients of the linear map. The values are randomly generated and are considered to be in *logarithm* form, because the private coefficients, linear map coefficient and vinegar values are directly multiplied from the first computation. Thus, storing values in *logarithm* representation is more efficient than leaving them in their *normal* representation when we consider the next operation. This method has one more advantage. In the *logarithm* representation, we can express the additional value of “0”, which does not exist in the *logarithm* look-up table, so it cannot be used in the *normal* representation. However, the value exists in the *exponential* table, so we can use this representation for private coefficients.

To find the inverse of a value, we can use an *inversion* table, which transforms the value using the *logarithm* table and then subtracts 0xff (255) before returning the resulting value to a *normal* representation. In our implementation, we use the modified *inversion* table described in Table A1, which outputs results in *logarithm* representation with input variables in normal representation. These directly multiply the inverse value in the Gaussian elimination process described in Algorithm 3, in which the first column is inverted and then multiplied with the remaining values in the same row, thus setting the first column to one. After that, each row is bit-wise exclusive-ORed with other rows. This process, called forward elimination, continues to generate triangular form. In the backward elimination, from the last row, the equation is solved by each row.



**Algorithm 3:** Multiplication algorithm using *logarithm* representation written in assembly where ADD, ADC and CLR is addition, addition with carry and clear and Rd and Rr are destination and source registers, (ADD Rd, Rr : Rd←Rd+Rr, ADC Rd, Rr: Rd←Rd+Rr+C, CLR Rd: Rd←Rd ⊕Rd, Rd: destination register, Rr: source register, *r*_1_ is cleared.)
Input: Unsigned bytes *A^l^*(*R*_2_), *B^l^*(*R*_3_)Output: Unsigned byte *A^l^*(*R*_2_) = *A^l^*(*R*_2_) + *B^l^*(*R*_3_)1.ADD *R*_2_, *R*_3_2.ADC *R*_2_, *R*_1_


### 3.2. Assembly Programming

Assembly programming generally exhibits higher performance than high-level programming, such as C and JAVA. This is because we can optimize register allocation and use the status register, which provides a carry bit to determine a certain condition. In our implementation, we adopt assembly programming throughout the whole process to reach to highest performance. Multiplication in *logarithm* representation can be simplified in addition by using the assembly described in Algorithm 3. First, register *r*_1_ is reset. Second, the operands are added. However, if the result is bigger than 0xff, an addition operation on the eight-bit microprocessor generates an output that is subtracted from 256, setting the carry bit. Therefore, this does not give the expected result. However, conducting an addition with the carry bit on the results, we can output the correct result, as we expected.

There is another case that exists. Algorithm 4 describes the exception condition. After central map computations, every parameter is stored into *normal* representation. The representation cannot map a zero variable into *logarithm* representation, so we should conduct exception handling for the zero variable. In this case, we directly output zero as a result without computation. In Algorithm 4, Step 1 clears destination register *R*_8_. From Steps 2 to 5, input variables (*R*_2_ and *R*_3_) are compared with zero (*R*_1_) to determine the zero variable. From Steps 6 to 13, *logarithm* mapping is conducted. In Steps 14 and 15, multiplication of *R*_2_ and *R*_3_ is conducted in *logarithm* representation. In Steps 16 to 19, mapping to *normal* representation is conducted.



**Algorithm 4:** Exception handling in multiplication for zero variables, where CP, BRCC and MOVW are compare, branch with carry and move register, (CP Rd, Rr: Rd-Rr, BRCC k: if (C=0) then PC ← PC+k+1, MOVW Rd, Rr: Rd+1:Rd ← Rr+1:Rr, ADD Rd, Rr: Rd←Rd+Rr, ADC Rd, Rr: Rd←Rd+Rr+C, CLR Rd: Rd←Rd ⊕Rd, Rd: destination register, Rr: source register, k: label, *R*_1_ is cleared, *R*_4_, *R*_5_ indicate *logarithm* table, *R*_6_, *R*_8_ indicate *exponential* table, *R*_28_, *R*_29_ indicate *Y* pointer, ZERO: label name.)
Input: Unsigned bytes *A^n^*(*R*_2_), *B*^n^(*R*_3_)Output: Unsigned byte *C^n^*(*R*_8_) = *A^n^*(*R*_2_) × *B^n^*(*R*_3_)1.CLR *R*_8_2.CP *R*_1_, *R*_2_3.BRCC **ZERO**4.CP *R*_1_, *R*_3_5.BRCC **ZERO**6.MOVW *R*_28_, *R*_4_7.ADD *R*_28_, *R*_2_8.ADC *R*_29_, *R*_1_9.LD *R*_2_, *Y*10.MOVW *R*_28_, *R*_4_11.ADD *R*_28_, *R*_3_12.ADC *R*_29_, *R*_1_13.LD *R*_3_, *Y*14.ADD *R*_2_, *R*_3_15.ADC *R*_2_, *R*_0_16.MOVW *R*_28_, *R*_6_17.ADD *R*_28_, *R*_2_18.ADC *R*_29_, *R*_1_19.LD *R*_8_, *Y*20.**ZERO:**


### 3.3. Vinegar Monomials

Central map computation is multiplying private coefficients with vinegar variables by the following equation: $f^{\left(k\right)}(u_{1},\ldots ,u_{n}):={\text{∑}}_{1\leq i\leq j\leq n}\gamma_{ij}^{\left(k\right)}u_{i}u_{j}=u^{T}\delta^{\left(k\right)}u$. These vinegar computations are executed in every central map computation, so vinegar monomials can reduce the vinegar variable computations throughout the whole processes by removing multiplication operations between each vinegar variable. Algorithm 5 describes the pre-computation of vinegar variables, in which we can generate vinegar monomials. The vinegar variables are generated in *logarithm* form. We left vinegar monomials as a *logarithm* form, because these variables are directly used for multiplication operations in ∑*_i_*_∈_*_V_*_,_*_j_*_∈_*_V_ γ_ij_p*_(_*_i_*_,_*_j_*_)_, where *γ* and *p* are the private coefficient and vinegar monomials, respectively.



**Algorithm 5:** Pre-computation of vinegar variables; symbols *V* and *n* denote the number of vinegar variables and the total number of vinegar and oil variables, respectively.
Input: Vinegar variables *u_i_*.Output: vinegar monomials *p*_(_*_i_*_,_*_j_*_)_, 0 < *i*, *j* < *V*.1.for *i* = 1 to *V* do2. for *j* = *i* to *V* do3.  *p*_(_*_i_*_,_*_j_*_)_ = *u_i_* × *u_j_*4. end for5.end for


### 3.4. On-the-Fly Computation



PKS requires a large key size. If we firstly compute all private keys before we use them, these occupy a huge storage amount for retaining these values. To avoid this condition, we selected the on-the-fly method, which generates private keys, and then, these keys are used directly. The AES-based PRNG that we chose generates 16 bytes of secret information every computation. These variables are directly used for central and linear map computation. For efficient computation, we divide central map computation into vinegar and oil parts. Firstly, the vinegar part is computing ${\text{∑}}_{1\leq i\leq j\leq \upsilon }\gamma_{ij}^{\left(k\right)}u_{i}u_{j}$ with vinegar coefficients, vinegar monomials and message variables, and then, the oil part is executing ${\text{∑}}_{1\leq i\leq \upsilon ,1\leq j\leq o}\gamma_{ij}^{\left(k\right)}u_{i}$ with vinegar and private coefficients.

### 3.5. Overview of the Computation Process

**Figure 3.**
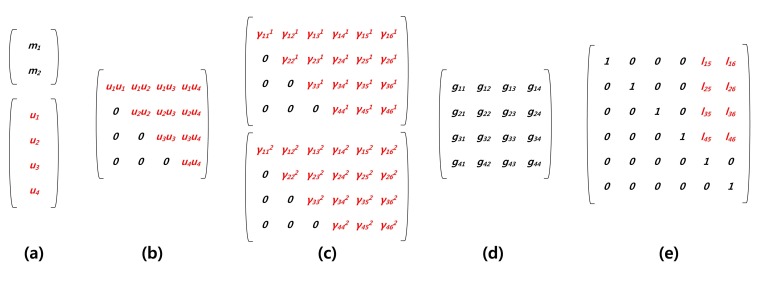
Overview of the representation of the variables for each computation: (**a**) message (*normal*), vinegar variables (*logarithm*); (**b**) vinegar monomials (*logarithm*); (**c**) private coefficients(*logarithm*); (**d**) Gaussian coefficient (*normal*); (**e**) linear map coefficients (*logarithm*); red and black mean *logarithm* and *normal* representations, respectively.

In Figure 3, briefly, we describe the representation of variables for each computation. Firstly, in Figure 3a, a message is hashed and outputted in *normal* representation. In the case of vinegar variables, *logarithm* representation is selected. In Figure 3b, ∑*_i_*_∈_*_V_*_,_*_j_*_∈_*_V_u_i_u_j_* are the vinegar monomials for efficient computation and are stored in *logarithm* form. In Figure 3c, we firstly compute ∑*_i_*_∈_*_V_*_,_ *_j_*_∈_*_V_γ_ij_p*_(_*_i,j_*_)_, where *γ* and *p* are private coefficient and vinegar monomials, respectively. After that, we compute the remaining part, ∑*_i_*_∈_*_V_*_,_*_j_*_∈_*_OV_γ_ij_u*_(_*_i,j_*_)_, to complete central map computation. In Figure 3d, Gaussian elimination is conducted with the results of the previous step. Finally, in Figure 3e, we generate linear map coefficients in *logarithm* representation and then compute the linear map computations.

## 4. Results

In this section, we provide evaluation results on a UOV scheme implementation in terms of memory consumption and computational complexity. Memory consumption is mainly used for key storage. The private key size is $o(o\upsilon +\frac{\upsilon (\upsilon +1)}{2})+o\upsilon$ for central map and linear map coefficients.

To compute the signature generation, previous implementations stored the private key in memory. However, our implementation stores only the seed values for the random number generator used for secret coefficient generation. For this reason, we can reduce the size of the private key by up to 99.9%. Furthermore, we show a performance enhancement by about 5.78% and 12.19% in signature generation and verification, respectively. This performance is achieved by adopting various optimization techniques that we explored before. The detailed evaluation results are available in Table 3 and 4, respectively.

### 4.1. Computational Costs

Table 5 shows a detailed analysis of the computation costs for each operation. We separate whole computations into six categories. In central map computation, we divide into vinegar and oil parts. In Gaussian elimination, we divide into forward and backward eliminations. Our method exploits the AES operation for PRNG, so computations that need many private coefficients take many clock cycles. In our implementation, central map computation is the most expensive operation, because the private coefficient size is *o*(*oυ*), and it needs 882 AES operations. This result could be reduced more, because we compute two AES operations (32 outputs) in each column for 28 coefficients and did not use four remaining outputs, due to the difficult variable handling. If we fully use 32 outputs, the speed would be improved further.

### 4.2. Source Code Storage (ROM) Requirements

Table 3 shows the reduction in size of the private key compared with traditional implementations. The size of the seed is computed with the following requirements (security level bit × 3, two for PRNG and one for the hash function). For all parameter variations, our implementation shows a 99.9% reduction in private key size. This is because our method generates private coefficients on the spot, so we store small seed values instead of whole private key values.

### 4.3. Variable Storage (RAM) Requirements

RAM stores persistent, counting or temporary variables for computation. The minimal RAM requirements are those for storing Gaussian elimination variables, which are generally of *o*^2^ complexity. For better performance, we used more RAM to compute the vinegar variables and look-up tables. The computed vinegar variables, which multiply two vinegar variables, are used several times in the central map, so maintaining these values is more beneficial than computing them each time. These have a size of $\frac{\upsilon (\upsilon +1)}{2}$. Second, the *logarithm*, *exponential* and *inversion* tables are used for converting the representations, and each table has a size of 256 bytes. By storing values in RAM, we can access data that is frequently required with lower overheads. The detailed information is available in Table 6.

### 4.4. Security Analysis

In this paper, we used representative block cipher AES as a core operation of random number generator and hash function to reduce the private key size. Therefore, the security levels highly rely on the strength of the block cipher. Recently, the vulnerability of a symmetric cryptosystem toward a quantum system has been proven by applying Grover's algorithm to break a symmetric algorithm by brute force, requiring 2*^n^*^/2^ of time, where *n* is the security bit [21,22]. For this reason, we should select a double-bit size to maintain the same security level of 

PK. This is the main strength of a quantum cryptosystem. To meet the *n*-bit security level of 

PK, we should select at least a 2*n*-security level. For this reason, we selected 128-bit AES encryption to meet the 64-bit security level of 

PK. To ensure the 96-, 128-bit security level, we could use 192-, 256-bit AES operations, which are also available in modern microprocessors. There is no specific conference and journal described.

### 4.5. Impacts on Other Protocols and Target Devices

We selected ATxmega128a1 as a target device to implement 

PKS. This does not mean that our method is limited to only the ATxmega128a1 board. There are only two requirements that exist for applying our methods. First, the microprocessor should support above an eight-bit word size, because our algorithm requires at least an eight-bit word size to use optimal *GF*(2^8^). Recently, eight-, 16-, 32-bit machines have been most widely used in embedded environments. The representative target devices in eight-, 16- and 32-bit are the XMEGA, MSP and ARM series, respectively. This means the majority of the embedded system could be improved by our methods. Second, the AES accelerator should be embedded in target devices. This requirement is also commonly met in modern microprocessors. Previously, we mentioned the representative target devices, including XMEGA, MSP and ARM v8, provide the AES accelerator as a peripheral.

In the case of this scheme, our method would have huge impacts on other 

PKS. We implemented the UOV scheme in this paper, presenting private key reduction methods. This could be applied to other schemes, including UOV, Rainbow and enTTS, without difficulty. Because these are variant of the UOV scheme, the key generation process is similar to the UOV scheme. Furthermore, this is not limited to the size of the finite field, because our method is generally ideal; so it could be extended to other fields, as well.

## 5. Conclusions

The majority of previous results focused on small public key 

PKS implementations. However, no practical results on the reduction of private key size in embedded microprocessors have been reported. In this paper, we presented a novel parallel computing method using a block cipher-based random number generator and a hash function to reduce the size of the private key and to boost speed performance. The method generates private coefficients, computing the central and linear maps simultaneously, because the AES accelerator embedded in modern microprocessors, including the ATxmega, MSP430 and ARMv8 series, can compute AES operations independently with the microprocessor. The results showed a significant reduction in private key size and enhancement in computation costs for signature generation and verification. These results can be applied to other schemes, such as Rainbow and enTTS, to generate private coefficients. Future work involves implementing this scheme on recent platforms, including Compute Unified Device Architecture (CUDA), Open Computing Language (OpenCL), NEON, Streaming SIMD Extensions (SSE) and Advanced Vector Extensions (AVX).

## Figures and Tables

**Table 1.** Result of NISTrandom number generator (RNG) test.

**Statistical Test**	**Proportion(%)**
Frequency	99
Block-Frequency	99.4
Cumulative-sums	99.2
Runs	99.5
Longest-run	100
Rank	100
FFT	100
Serial	100
Lempel–Ziv	100
Linear-complexity	100

**Table 2.** Speed of compression functions.

**Algorithm**	**Time (cycles/byte)**	**RAM (bytes)**	**ROM (bytes)**
SHA-1 [17]	579	198	1,022
SHA-1 [18]	177	122	1,352
SHA-256 [17]	783	416	1,598
SHA-256 [18]	335	158	2,720
Blake-32 [17]	1,115	245	6,684
Blake-32 [19]	324	251	1,804
Blake-32 [18]	263	206	2,076
Skein-256 [18]	287	123	2,464
Ours (Davies *et al.*)	50	48	144

**Table 3.** Implementation results of the signature generation of the UOVscheme.

**Scheme**	**Private Key (Byte)**	**Cycles** ×10^3^	**Time (ms) 32 MHz**	**ROM (Byte)**	RAM **(Byte)**	**Program Language**
UOV (21,28) [20]	21,462	1,615	50.49	2,188	441	C + ASM
0/1 UOV (21,28) [20]	21,462	1,577	49.29	2,258	441	C + ASM
Our UOV (21,28)	**48**	**1,486**	**46.37**	**4,814**	**2,499**	**ASM**

**Table 4.** Implementation results of the signature verification of the UOV scheme.

**Scheme**	**Private Key (Byte)**	**Cycles** × 10^3^	**Time (ms) 32 MHz**	**ROM (Byte)**	RAM **(Byte)**	**Program Language**
UOV (21,28) [20]	25,725	1,690	52.83	466	n/a	C + ASM
0/1 UOV (21,28) [20]	4,851/20,874	1,395	43.60	578	n/a	C + ASM
Our UOV (21,28)	**25,725**	**1,225**	**38.30**	**2,069**	**562**	**ASM**

**Table 5.** Computation costs for each operation in the case of UOV (21,28) on the ATxmega128a1.

**Operation**	**Clock Cycle**	**Proportion (%)**	**Number of AES Operation**
Vin-genand pre-com	9,396	0.63	2
Central-vinegar	514,070	34.59	546
Central-oil	**769,107**	**51.75**	**882**
Gaussian-forward	128,253	8.69	-
Gaussian-backward	1,460	0.09	-
Linear	62,896	4.23	42
Total	1,486,182	100	1,367

**Table 6.** Minimal and our RAM requirements Minimal and our RAM requirements in bytes.

**Scheme**	**UOV (21,28)**	**UOV (28,37)**	**UOV (44,59)**	**General**
Minimal	441	784	1,936	*o*^2^
Our	1,615	2,255	4,474	$o^{2}+\frac{\upsilon (\upsilon +1)}{2}+3(16\times 16)$

## Appendix

### Inversion Table

Table A1 describes the inversion look-up table on *GF*(2^8^). I = The input is the *normal* representation, but the output value is the inverse of the input in *logarithm* representation form.

**Table A1.** Inversion table (input: *normal* representation, output: *logarithm* representation).

	0	1	2	3	4	5	6	7	8	9	a	b	c	d	e	f
0	—	00	e6	fe	cd	fd	e5	39	b4	38	e4	97	cc	11	20	fc
1	9b	fb	1f	f1	cb	72	7e	10	b3	8e	f7	37	07	96	e3	3e
2	82	3d	e2	4a	06	46	d8	95	b2	1b	59	8d	65	36	f6	87
3	9a	d0	75	fa	de	f0	1e	db	ed	0f	7d	ba	ca	6c	25	71
4	69	70	24	42	c9	2f	31	6b	ec	a3	2d	0e	bf	b9	7c	c7
5	99	22	02	cf	40	f9	74	9d	4c	da	1d	67	dd	77	6e	ef
6	81	91	b7	3c	5c	49	e1	bd	c5	94	d7	ab	05	7a	c2	45
7	d4	86	f5	ea	64	60	a1	35	b1	2b	53	1a	0c	8c	58	a8
8	50	a7	57	af	0b	15	29	8b	b0	51	16	2a	18	19	52	17
9	d3	28	8a	85	14	e9	f4	0a	a6	34	a0	4f	63	56	ae	5f
a	80	f3	09	90	e8	3b	b6	13	27	bc	e0	d2	5b	89	84	48
b	33	44	c1	a5	04	9f	4e	79	c4	ad	5e	93	55	aa	d6	62
c	68	4d	78	6f	9e	41	23	03	43	6a	30	32	c8	c0	a4	2e
d	ac	c6	7b	c3	be	5d	92	b8	eb	d5	61	a2	a9	0d	2c	54
e	bb	ee	6d	26	dc	df	d1	76	4b	83	47	d9	88	66	1c	5a
f	98	b5	12	21	3a	ce	01	e7	f2	9c	73	7f	3f	08	8f	f8
